# Supplementary material for: A cohort study on the association between changing occupational stress, hair cortisol concentration, and hypertension
Source: PLoS One. 2023 May 17;18(5):e0285623. doi: 10.1371/journal.pone.0285623 (PMC10191276; doi:10.1371/journal.pone.0285623)
Supplement: Attachment — Submitted filename: Response to Reviewers.docx [file pone.0285623.s001.docx]

Response to Reviewers

Thank you for your very valuable comments and useful suggestions. In response to your suggestions, we do the following point-to-point responses. With your comments and help, we believe that the quality of our articles will be greatly improved.

Reviewer #1:

1) My main concern is that by categorising the primary exposure and outcome variables (an approach that is not well explained), the authors are losing valuable information. This is particularly prolematic for the primary predictor, why not look at scores at baseline follow-up and their interaction rather than increase/decrease/stable. Moreover, categorisation poses problems for some variables due to small cell sizes - for example - in Table 4 the number of cases who developed hypertension are 9, 2, and 10 in the lower HCC groups. The categorisation of is particularly unclear for blood pressure, is a change in absolute levels classed as a decrease/increase or was a threshold for amount of change required? So few people classed as invariant implies that this might difference in absolute levels which would be highly inappropriate. Further details required.

**Response 1)：**We were very sorry for the confusion and have elaborated the basis for division in the revised manuscript. (Page 9, Line 129) According to Yang XW’s [1] method of developing occupational stress norms and conversion tables for managers, ORQ scores were transformed into t scores to represent the degree of occupational stress, and t scores were graded. We have explained in the revised manuscript. (Page 9, Line 139-145) The rules for identifying changes in occupational stress during follow-up are shown in the table below.

|  | **follow-up occupational stress** | | | |
| --- | --- | --- | --- | --- |
| **baseline occupational stress** | High | middle | normal | lack of |
| High | - | ↓ | ↓ | ↓ |
| middle | ↑ | - | ↓ | ↓ |
| normal | ↑ | ↑ | - | ↓ |
| lack of | ↑ | ↑ | ↑ | - |

↑：increase

↓：decrease

-：stable

As the sample size of workers who collected hair in this study did not reach the minimum theoretical sample size, this limitation has been mentioned in the Discussion section of the revised manuscript. (Page 23, Line 310-312)

As in the study of Stalder T et al.[2], even if the value of blood pressure did not increase to the threshold, Elevated systolic and diastolic blood pressure were also associated with HCC.

1.	Yang XW, Wang ZM, Jin TY, Lan YJ. [Study of the occupational stress norm and it's application for the executive group and administrative support group]. Wei sheng yan jiu = Journal of hygiene research. 2006;35(4):477-80. Epub 2006/09/22. PubMed PMID: 16986528.

2.	Stalder T, Steudte-Schmiedgen S, Alexander N, Klucken T, Vater A, Wichmann S, et al. Stress-related and basic determinants of hair cortisol in humans: A meta-analysis. Psychoneuroendocrinology. 2017;77:261-74. Epub 2017/01/31. doi: 10.1016/j.psyneuen.2016.12.017. PubMed PMID: 28135674.

2) Statistical analysis section is unclear, terms are used that are not described and it is not clear how these relate to the variables examined. Stepwise regression is mentioned in the final sentence, not clear whether this is forward/backward or how threshold for inclusion determined. Stepwise models are problematic as they often yield inconsistent results depending on the decisions above, instead, deciding a priori whether confounders should be included is more appropriate.

**Response 2)：** We felt sorry for the confusion. We used enter methods to build two logistic regression models and conduct mediation effect analysis. We have revised and supplemented it in the methods of the section revised manuscript. (Page 12, Line 195-204)

3) Abstract: please add cohort details in methods or results (e.g., mean age, percentage male). Methods slightly unclear.

**Response 3):** We would like to thank the reviewer for the comment and we have added it in Methods section of the manuscript. (Page 2, Line 8-9)

4) Methods: the description of the occupational stress questionnaire could be clearer. Why was this subscale selected specifically are the other scales not relevant?

**Response 4):** We felt sorry for the confusion, the Revised Occupational Stress Inventory (OSI-R) scale included three subscales: Occupational Role Questionnaire (ORQ), Personal Strain Questionnaire (PSQ), and Personal Resources Questionnaire (PRQ). The ORQ reflects the level of occupational stress experienced by individual workers, the PSQ reflects the individual stress response, and the PRQ reflects the ability to cope with stress response, so we chose to use the ORQ scale. [3].

3.	J L, YJ L, ZM W, MZ W, MZ W, GQ L. The test of occupational stress inventory revised edition. Chinese Journal of Industrial Hygiene and Occupational Diseases. 2001;03:34-7.

5) Methods: Was the hair sample collected from the root (ie plucked) or was it cut as close to the root as possible?

**Response 5):** We collected 20-30mg hair samples from the 2 ~ 3cm of the root of the subject's hair.

6) Results: the number of results tables is overwhelming, can those with the same outcome or predictor variable be combined?

**Response 6):** We would like to thank the reviewer of this suggestion and we have merged the original Table 1 and Table 3 into the current table 1, and the original table 2 and Table 4 into the current table 2 in the revised manuscript.

7) Results: Chi squared test adds nothing but the descriptives show that the numbers with outcome are small for reference category

**Response 7):** Thank you for your question. We have modified the tables of the chi-square tests in the results (Tables 1 and 2).

8) Table 7: if p indicates P value then values of 0 should be replaced with p<0.001

**Response 8):** We felt grateful for the comment and we have revised it in Table 7.

9) Discussion: Readability of the first paragraph of the discussion could be improved - sentences could flow better.

**Response 9):** Thanks for the informative comment. We have modified this paragraph in the discussion.

10) Discussion, line 235 - do the authors mean 'mental health workers' instead of 'mental workers'?

**Response 10):** We felt very sorry for the confusion We have revised it by white-collar (line 261) in the revised manuscript.

11) Overall the discussion could be more succinct but should also acknowledge studies which show contrasting findings to those observed in the current study.

**Response 11):** We felt grateful for the suggestion and we have revised it in the revised manuscript according to your suggestion.

12) Discussion includes nothing on the proposed mechanisms

**Response 12):** We would like to thank the reviewer of this suggestion and we have we have elaborated the mechanisms in the Discussion.

13) There are typographical errors in the text, in particular whole words that have been hyphenated e.g., line 27 'dis-ease'

**Response 13):** We apologized for the editing error and we have revised it in the revised manuscript.

Reviewer #2:

This manuscript was a very interesting read to me as I have been working on work stress and cortisol research over the last two years. Correlating hair cortisol concentration with hypertension and work stress was a great idea for a scientific manuscript. The statistical analyses appear accurate and sound and the manuscript as a whole is nearly publishable.

The list below includes any concerns I had with the manuscript as it currently stands. Many of them are grammatical in nature. Please be sure to not write in first person (using "we") in scientific literature and don't confuse correlation with causation.

**Response:** Thanks for the informative comment. We are honored to receive such a high evaluation. The questions you raised about grammar and first person have been revised in the article, and the correlation and causation have been clarified. We will further improve the manuscript and strive to meet higher requirements.

Reviewer Notes

14) Line 27: No reason to have the hyphen in disease.

15) Line 29: Remove the period after “[4]”

**Response 14-15):** We were very sorry for the mistakes and we have corrected it in the revised manuscript.

16) Line 43: Please be more specific than saying “workers” work in traditionally high-pressure occupations.

**Response 16):** We have elaborated it specifically in the revised manuscript. (Page 5, Line 47)

17) Line 45: Unsure what you mean by “mental workers”.

**Response 17):** We felt very sorry for the confusion. We have revised it by white-collar (line 49) in the revised manuscript.

18) Line 52: No need to have a hyphen in researchers

19) Line 70: Change “Compared with in blood…” as you have two prepositions back-to-back. I’d suggest “Compared with cortisol in blood…”

20) Line 88: Don’t write in first person.7

21) Line 90: Add a comma after “study”

Great sample size

22) Line 112: No reason for hyphen in subjects

**Response 18-22):** We felt very sorry for the error and we have corrected in the revised manuscript.

23) Line 155: Can you better define “irregular” physical exercise?

**Response 23):** We felt sorry for the confusion and we have clarified in the manuscript. (Page 11, Line 175)

24) Line 156: Can you distinguish “no alcohol consumption” vs “abstinence”?

**Response 24):** We felt sorry for the confusion. “no alcohol consumption” here means never drinking, while “abstinence” means having a history of drinking but not drinking now.

25) Line 157: Can you describe “smoking cessation”?

**Response 25):** We felt sorry for the confusion, “smoking cessation”referred to the subjects used to smoke but do not smoke now. We have replaced smoking cessation in the article with “ex-smoker”. (Page 11, Line 178)

26) Line 223 & Line 224: change “level” to “levels”

27) Line 225: Don’t write in 1st person

**Response 26-27):** We apologized for the editing error and we have modified it in the revised manuscript.

28) Line 232: Move the short paragraph beginning in line 230 into the next paragraph that begins on line 235. Additionally, the two sentences in lines 230-234 are pretty redundant in purpose. I’d suggest editing these two sentences in some way.

**Response 28):** We have adjusted it in the revised manuscript. (Page 20, Line 255-260)

29) Line 235: What are “mental workers”?

**Response 29):** “mental workers” means people who rely on their brains to work. Now we have changed the text from mental workers to white-collar (line 265).

30) Line 239: Don’t end a sentence in a helping verb “was”.

31) Line 256: No need for the hyphen in the word service.

32) Line 278: No need for the hyphen in the word under.

33) Line 305: No need for the hyphen in the word required.

34) Line 308: Don’t write in 1st person.

**Response 30-34):** We were so sorry for these mistakes and we have revised it in the revised manuscript.

35) Line 309 and Line 312: You can’t necessarily say occupational stress caused high HCC (Line 309) or that occupational tension caused high blood pressure (Line 312). You can simply state that they were correlated with one another.

**Response 35):** We would like to thank the reviewer for the suggestion and we have modified it in the revised manuscript. (Page 24, Line 321-322)

36) Line 312: No need for the hyphen in the word increase.

**Response 36):** The hyphen in the word have been deleted in the revised manuscript.
