# Supplementary material for: A cohort study on the association between changing occupational stress, hair cortisol concentration, and hypertension
Source: PLoS One. 2023 May 17;18(5):e0285623. doi: 10.1371/journal.pone.0285623 (PMC10191276; doi:10.1371/journal.pone.0285623)
Supplement: Attachment — Submitted filename: Response to Reviewers.docx [file pone.0285623.s002.docx]

Response to Reviewers

Thank you for your very valuable comments and useful suggestions. In response to your suggestions, we do the following point-to-point responses. With your comments and help, we believe that the quality of our articles will be greatly improved. We have reviewed our reference list, revised the format and changes to the reference list have been highlighted in the revised manuscript.

Reviewer #1:

The revised paper is easier to follow and the authors have mostly addressed the grammatical issues, one remaining issue is that the authors often refer to 'white collar' rather than 'white collar workers' this needs to be addresed before publication.

**Response：**We felt grateful for the suggestion and we have revised it in the revised manuscript according to your suggestion. (Page 5, Line 51;Page 20, Line 260)

Reviewer #2:

The only last concern I have is that the "no alcohol consumption" vs "abstinence" still hasn't been addressed within the manuscript itself. As long as this difference can be stated, I think the manuscript should move on towards publication.

**Response:** Thanks for the comment. We will further improve the manuscript and strive to meet higher requirements. We have revised it in the revised manuscript. (Page 11, Line 177；Table1)
